# Data to support observation of late and ultra-late latency components of cortical laser evoked potentials

**DOI:** 10.1016/j.dib.2015.11.021

**Published:** 2015-11-23

**Authors:** Andrej Stancak, Stephanie Cook, Hazel Wright, Nicholas Fallon

**Affiliations:** Department of Psychological Sciences, University of Liverpool, Liverpool L69 7ZA United Kingdom

**Keywords:** Pain, Evoked potentials, EEG, Source analysis

## Abstract

Data are provided to document the presence of late and ultra-late latency components of cortical laser evoked potentials (LEPs) following noxious laser stimulus in Stancak et al. (2015) [3]. The latency components, labeled provisionally as N4, N5, and N6, were observed in 16 healthy human participants who were asked to fully attend their painful and non-painful sensations occurring in association with noxious laser stimulus. Individual laser evoked potential waveforms are provided in support of this observation. Data provided demonstrate the cortical sources of the late and ultra-late laser evoked potentials. The cortical sources of LEPs were reconstructed using the standardized Low Resolution Electromagnetic Tomography (sLORETA) method.

**Specifications Table**

**Table:** 

Subject area	*Neuroscience*
More specific subject area	*Pain*
Type of data	*Figures*
How data was acquired	*Electroencephalography; evoked potentials; source dipole analysis*
Data format	*Individual evoked potential waveforms in an ASCII file. Statistical T-maps of cortical activation in the form of sLORETA maps*
Experimental factors	*Multifactorial design involving factor analysis and multivariate regression analysis*
Experimental features	*Single-trial laser-evoked potentials acquired in 16 subjects were correlated with subjective dimensions (factors) of pain experience*
Data source location	*Liverpool, United Kingdom*
Data accessibility	*Data are provided with this article*

**Value of the data**

• Data allow a comparison with laser evoked potentials curves acquired in chronic pain patients.
• Source activation maps data can be added to the list of cortical sources of laser evoked potentials in a meta-analysis study.
• Data can be used to postulate novel hypotheses about the origin of late and ultra-late latency components during cortical processing of nociceptive input.

## 1 Data

### 1.1 Individual laser evoked potentials

**Fig. 1:**
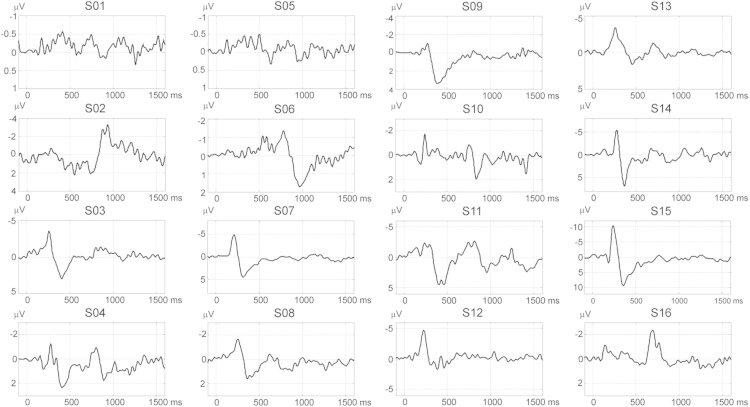
Individual LEP waveforms at electrode Cz or a neighbor midline electrode in 16 subjects (S01–S16). The waveforms demonstrate, with variable amplitudes, the presence of late and ultra-late latency components in the latency range 700–940 ms and 1000 ms.

The latency components of LEPs in the range of 120–180 ms (N1), 180–280 ms (N2), and 280–400 ms (N3/P2) were seen in a number of previous studies. Recently, late LEP components denoted as C-N2 and C-P2 ranging up to the latency of 940 ms have been reported [1]. We present data confirming the presence of novel LEP components in the latency range 700–940 ms, and an additional latency component in the ultra-late latency range >1000 ms. Data in Fig. 1 show the presence of long- and ultra-long latency components at the vertex electrode Cz or a neighbor midline electrode in 16 healthy participants. The amplitudes of LEP components show a large inter-individual variability. LEP waveforms of all 16 subjects are provided in a supplementary file which contains ASCII data as a matrix sized 426 time samples×16 subjects.

### 1.2 Source activation maps of individual latency components

**Fig. 2:**
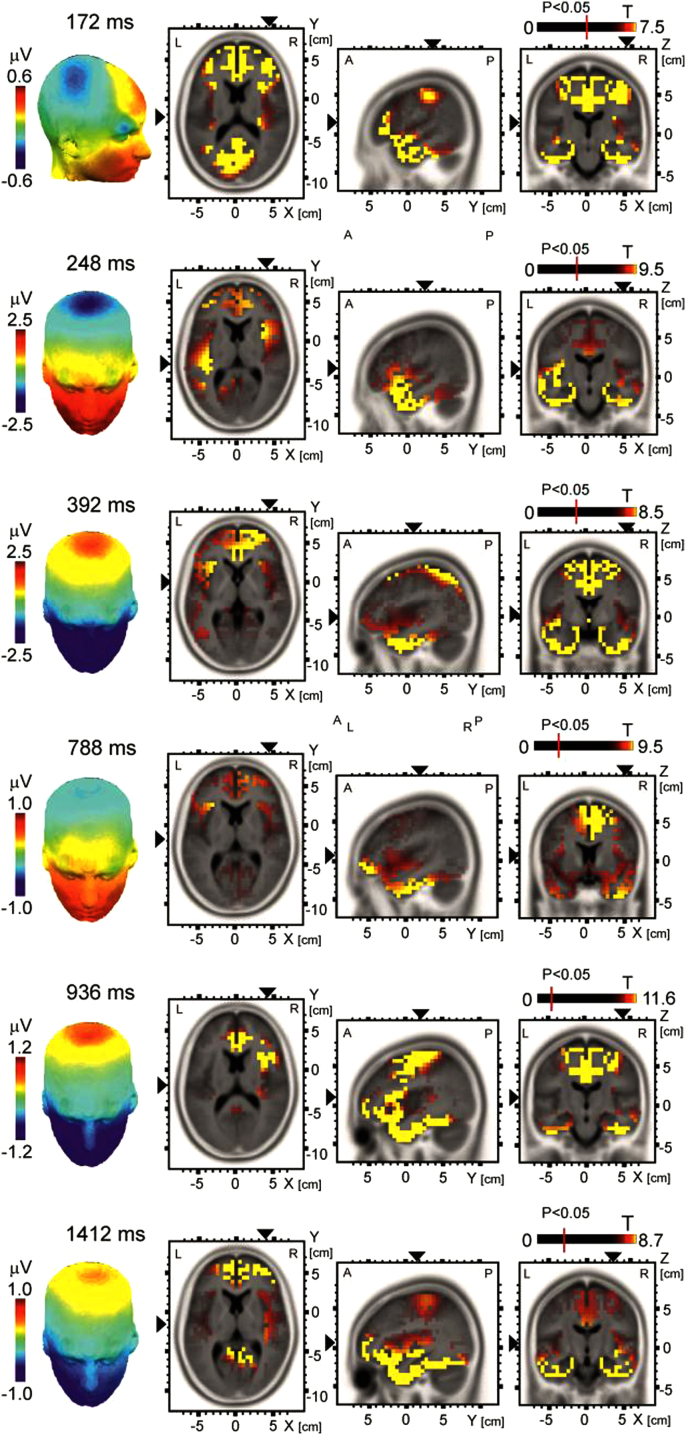
The LEP potential maps (left panel) and sLORETA T-maps (right panel) in select latency components (N1 – 172 ms, N2 – 248 ms, N3/P2 – 392 ms, N4 – 788 ms, N5 – 936 ms, N6 – 1412 ms). Maps were scaled to 60–75% of the maximum *T*-value. The vertical red bar crossing each scale indicates the *T*-value at a corrected *P* value of 0.05. The sLORETA maps show cortical activations in transversal (left map), sagittal (middle map), and axial (right map) planes. The slices viewed are indicated by black triangles. R=right, L=left, A=anterior, P=posterior.

Activation maps of previously known LEP components (N1 (172 ms), N2 (248 ms) and N3/P2(392 ms), and of the novel LEP components N4 (768 ms), N5 (936 ms), and N6 (1412 ms) are shown in the form of potential maps (LEPs) and statistical T-maps computed using individual sLORETA maps of 16 subjects (Fig. 2). The statistical T-maps for each of six latency components shown in Fig. 2 are supplied in the form of binary (*.slor) files for viewing in sLORETA program.

## 2 Experimental design, materials and methods

LEPs were recorded using a 129-channel Geodesic EGI system (Electrical Geodesics, Inc., Eugene, Oregon, USA) with 1 kHz sampling rate in 16 healthy participants. Sixty laser stimuli were applied to the dorsum of the left hand. Subjects were asked to fully attend any painful or non-painful sensations associated with laser stimulus, and rate their sensations using a set of visual analogue scale. EEG data were cleaned in BESA v. 6.0 program (Megis GmbH, Munich, Germany), and pruned further using Independent Component Analysis in EEGLab v. 11.04.3b (http://sccn.ucsd.edu/eeglab/). The filters were set to 0.5–30 Hz. EEG data were segmented into epochs ranging from −300 ms to 1600 ms relative to onset of laser stimulus, and averaged. The baseline period ranged from −300 to 0 ms. Data were down-sampled to 250 Hz for the purpose of statistical analysis.

Source localization analysis was performed in selected, averaged, 20-ms time intervals using sLORETA [2], implemented in LORETA-Key v. 200840–403 program (www.keyinst.unizh.ch/loreta). sLORETA evaluates distributed electrical sources by smoothing the inverted images using a Laplacian smoothing operator. Source activation maps were computed in a grid of 6239 voxels sized 5×5×5 mm^3^, covering the whole cortical mantle. The regularization parameter, related to the signal-to-noise ratio, was set to 1.0. sLORETA maps in the selected time interval of 20 ms durations each, centered at the latency peaks of LEP components were computed in each of 16 subjects. The set of 16 sLORETA images was analyzed using a univariate *T*-test. The resulting T maps were assigned a threshold at a corrected *P* level of 0.05 using a permutation method involving 5000 randomizations. Further details on methods and results can be found in the accompanying article [3].
